# Impact of Oral Health Behaviors on Dental Caries in Children with Intellectual Disabilities in Guangzhou, China

**DOI:** 10.3390/ijerph111011015

**Published:** 2014-10-22

**Authors:** Zifeng Liu, Dongsheng Yu, Wei Luo, Jing Yang, Jiaxuan Lu, Shuo Gao, Wenqing Li, Wei Zhao

**Affiliations:** 1 Faculty of Medical Statistics and Epidemiology, School of Public Health, Sun Yat-sen University, Guangzhou 510080, China; E-Mail: sumsliu@hotmail.com; 2 Guangdong Provincial Key Laboratory of Stomatology, Department of Pediatric Dentistry, Guanghua School of Stomotology, Hospital of Stomatology, Sun Yat-sen University, Guangzhou 510055, China; E-Mails: yudsh@mail.sysu.edu.cn (D.Y.); luo_wei1988@126.com (W.L.); yang_jing1989@126.com (J.Y.); lujiaxuan1989@126.com (J.L.); gshuo@mail3.sysu.edu.cn (S.G.); wendysums@hotmail.com (W.L.)

**Keywords:** dental caries, prevalence, intellectually disabled child, oral health behavior

## Abstract

Dental care is consistently reported as one of the primary medical needs of children with disabilities (IDC). The aim of the present study was to explore the influence of oral health behaviors on the caries experience in children with intellectual disabilities in Guangzhou, China. A cross-sectional study was carried out in 477 intellectually disabled children, 12 to 17 years old, who were randomly selected from special educational schools in Guangzhou. A self-administered parental questionnaire was used to collect data on socio-demographic characteristics and oral health behavior variables, and 450 valid questionnaires were returned. Multiple regression analysis was used to examine the factors associated with dental caries. The average age of those in the sample was 14.6 years (SD = 1.3), 68.4% of whom were male, and the caries prevalence rate was 53.5% (DMFT = 1.5 ± 2.0). The factors significantly affecting the development of dental caries in IDC included gender, the presence or absence of cerebral palsy, and the frequency of dental visits and toothbrushing. In conclusion, the presence of cerebral palsy contributed to an increase risk of caries experience in intellectually disabled children, while toothbrushing more than twice a day and routine dental visits were caries-protective factors. Oral health promotion action may lead to a reduction in dental caries levels in IDC.

## 1. Introduction

Children with disabilities are at high risk for health problems [1]. In particular, children with intellectual disabilities (IDC) usually need support to carry out daily self-care. The level of self-care may be said to be directly related to the degree of learning disability and children with more profound impairments may need greater levels of support [2,3]. So, IDC usually require extra help and rely on their parents or guardians to achieve and maintain good health. Oral health is no exception. Dental care is consistently reported as one of the primary medical needs of children with disabilities [4]. Difficulties in communicating oral health needs and concomitant diseases (cerebral palsy, Down syndrome, *etc.*) can be barriers to adequate oral care and put the IDC at greater risk for developing oral health problems [5]. Several studies have reported a greater prevalence of caries in children with disabilities compared with that in the general population [6,7,8]; therefore, these children should obtain special preventive care in the dental office [9]. According to the latest report, there are 2.46 million children with an intellectual or physical disability in China, 30.9% of whom are IDC, which ranks first [10]. However, little attention has been paid to the oral health status of IDC, and still less information on their prevalence of dental caries and DMFT (Decayed, Missing due to caries, and Filled Teeth) scores is available in Mainland China. Thus, studies on the dental health of IDC are urgently needed.

Factors related to the dental health of IDC have been discussed in previous literature. Socio-demographic characteristics (age, gender, and disability), socio-economic indicators [11], and oral health behaviors may be related to the development of dental caries. A study by Moreira *et al.* indicated that children with both cerebral palsy (CP) and intellectual disabilities presented more dental cavities than controls, and that intellectual disability affected the development of dental caries [12]. Bakry and Alaki reported that age, gender, and parents’ education did not have a profound impact on caries experience in either children with intellectual disability or healthy children, while in IDC, the nature of diet and the severity of intellectual disability were significant risk factors associated with dental caries experience [13]. In Mainland China, however, the influence of risk/protective factors on the oral health of IDC has not yet been investigated. Because of significant differences in living habits and geographic location, whether the same factors in other regions would have the same effect on Chinese IDC is questionable. Therefore, we conducted this cross-sectional survey on the prevalence of caries and DMFT scores in IDC in Guangzhou city, aiming to explore the relationship between oral health behaviors and dental caries in IDC.

## 2. Methods

### 2.1. Study Design, Population and Sample

This study conservatively estimated caries prevalence in IDC to be 50.0%. Standard error was set at 5.0%, and the response rate was assumed to be 90.0%. At least 427 children were needed for study validity.

Our study was reviewed and approved by the Ethical Review Committee of the Institute of Stomatological Research, Sun Yat-Sen University, before the study began. The approval number is ERC-2012-028.

The present cross-sectional study was conducted among individuals from special educational schools in Guangzhou in March and April of 2013. Stratiﬁed by the numbers of schools and of the IDC enrolled, a representative sample of 6 special educational schools with 551 children was randomly selected. Schoolchildren were selected if they met the inclusion and didn’t have any exclusion criteria. They were included if they were aged between 12 and 17 years old and had a clinical diagnosis of intellectual disability based on the criteria and classification established by Standardization Administration of the People’s Republic of China [14], as registered in their medical records. We excluded children from the study for the reason that their parents refused to allow them to undergo the dental examination. The parents of each child were contacted and informed in writing about the study design and dental examination proposed for their children. If they agreed to have their children participate in the survey, a document of informed consent was voluntarily signed by the parents. In total, 477 IDC were recruited in the final sample.

### 2.2. Data Collection and Study Variables

The children’s DMFT scores were based on clinical examinations performed by two well-trained and calibrated dental practitioners (kappa = 0.87) [15]. The DMFT score has been well established as the key measure of caries experience in dental epidemiology, reflecting the degree of caries experience, and is calculated by adding results for Decayed, Missing due to caries, and Filled Teeth in the permanent dentition. Visual examination by means of an artificial white light source and plane mouth mirror was combined with probing diagnosis in caries examination, with codes and criteria as established by the WHO [16]. The information on grade of intellectual disability and the presence or absence of cerebral palsy was supplied by the special education school. Every child with a disability had been examined and assessed by the designated and qualified hospital. The results of the assessment were registered in the school’s medical records.

A self-administered parental questionnaire (11 items) was used to collect data on socio-demographic characteristics and oral health behavior variables. Socio-demographic variables included the gender of the children, parental education level, registered residence, and school district. The parental education level was categorized into “lower education” (1–12 years), “‘medium education” (13–15 years), and “higher education” (16 or more years) [17]. Grade of intellectual disability and the presence/absence of cerebral palsy were based on the criteria established by Standardization Administration of the People’s Republic of China and WHO respectively [14,18]. Oral health behavior variables, as used in previous research, included gargling habits after meals, dental visits in the preceding 12 months, frequency of toothbrushing, eating snacks, and eating sweet foods before sleeping [17,19,20,21]. In total, 477 questionnaires were delivered, and 450 valid questionnaires were returned, for a return rate of 94.3%.

### 2.3. Statistical Analysis

Statistical analysis was conducted with SPSS 13.0 for Windows, with the level of significance set at 5.0% (*p*-value < 0.05). Frequencies, means, standard deviations (SD), and percentages were computed for descriptive purposes. We used the Kolmogorov-Smirnov (KS) test to determine the distribution of DMFT scores. Since non-normal distribution was found, non-parametric tests (Wilcoxon rank sum test and Kruskal-Wallis H test) were used. Pearson's chi-square test was used to analyze the differences in the distribution of socio-demographic and oral health behavior variables (independent variables) between the case group (DMFT > 0) and the control group (DMFT = 0), and crude odds ratios (OR’s) and 95% confidence intervals (95% CI) were estimated. Unordered categorical variable (school districts) is defined as dummy viable with the method of reference cell coding. For the lowest prevalence rate of caries experience in our survey, Liwan was chosen as the reference cell, which is compared with all other districts. Variables that were significantly associated with DMFT scores were selected in the final model. Multivariate logistic regression analysis (Forward LR procedure) was conducted to investigate which independent variables were significant for explaining the dental condition.

## 3. Results

### 3.1. Description of the Study Sample

The average age of the children was 14.6 years (SD = 1.3), and 68.4% were male. The sample consisted of 450 children (241 DMFT > 0, 209 DMFT = 0), and the caries prevalence rate was 53.5% (DMFT = 1.5 ± 2.0). Table 1 displays the distribution of socio-demographic characteristics, oral health behavior variables, and DMFT scores (mean ± SD). A statistically significant difference in DMFT scores was detected between subgroups stratiﬁed by gender, the presence/absence of cerebral palsy, dental visit in the preceding 12 months, and frequency of toothbrushing, respectively (*p* < 0.05).

**Table 1:** Distribution of socio-demographic characteristics, oral health behaviors, and DMFT of children with intellectual disabilities.

Variables	*N* (%)	DMFT	
		(Mean ± SD)	*p* ^a^
**Socio-demographic Characteristics**			
Gender			0.002
Male	308 (68.4)	1.4 ± 1.9	
Female	142 (31.6)	1.9 ± 2.1	
Father’s education level			0.595
Low	92 (20.4)	2.45 ± 3.98	
Medium	248 (55.1)	1.73 ± 2.64	
High	110 (24.4)	2.11 ± 2.96	
Mother’s education level			0.468
Low	137 (30.4)	2.16 ± 3.13	
Medium	255 (56.7)	1.85 ± 3.00	
High	58 (12.9)	2.03 ± 3.06	
Domestic economic status			0.938
Low-income	25 (5.6)	1.5 ± 1.9	
Middle-income	312 (69.3)	1.6 ± 2.0	
High-income	113 (25.1)	1.5 ± 2.0	
Registered residence			0.125
Countryside	60 (13.3)	2.0 ± 2.2	
City	390 (86.7)	1.5 ± 2.0	
School district			0.136
Liwan	80 (17.8)	1.4 ± 2.0	
Hizhu	89 (19.8)	1.5 ± 1.9	
Yuexiu	93 (20.7)	1.2 ± 1.7	
Tianhe	90 (20.0)	2.1 ± 2.3	
Panyu	98 (21.8)	1.6 ± 2.0	
Grade of intellectual disability			0.470
I	33 (7.3)	1.3 ± 1.8	
II	82 (18.2)	1.6 ± 2.2	
III	182 (40.4)	1.7 ± 2.0	
IV	153 (34.0)	1.4 ± 1.9	
With cerebral palsy			0.008
No	295 (66.7)	1.4 ± 1.9	
Yes	155 (33.3)	1.8 ± 2.1	
**Oral Health Behaviors**			
Gargling after dinner			0.746
No	341 (76.7)	1.5 ± 1.9	
Yes	109 (23.3)	1.6 ± 2.1	
Dental visits in preceding 12 months			<0.001
No	344 (76.4)	1.7 ± 2.0	
Yes	106 (23.6)	1.0 ± 1.8	
Frequency of toothbrushing			<0.001
≤Once a day	306 (68.0)	1.8 ± 2.1	
>Once a day	144 (32.0)	1.0 ± 1.5	
Eating snacks			0.425
Occasionally or often	363 (80.7)	1.6 ± 2.0	
Never or seldom	87 (19.3)	1.3 ± 1.8	
Eating sweet foods before sleeping			0.173
Occasionally	65 (14.4)	1.9 ± 2.2	
Never	385 (85.6)	1.5 ± 2.0	

Notes: ^**a**^ Wilcoxon rank sum test or Kruskal–Wallis tests. *p* < 0.05 is considered statistically significant; DMFT: Decayed, Missing due to caries, and Filled Teeth; SD: standard deviation.

### 3.2. Single-Factor Analysis

Table 2 shows the socio-demographic results of single-factor analysis. No differences were observed in the variables of parents’ education, domestic economic status, registered residence, grade of intellectual disability, and school district between children with DMFT > 0 and those with DMFT = 0 (*p* > 0.05). Crude OR’s show that being female (OR = 1.8, *p* < 0.05, 95% CI = 1.2–2.7) and having cerebral palsy (OR = 1.6, *p* < 0.05, 95% CI = 1.1–2.4) were associated with an increased likelihood of caries experience.

Table 3 shows the oral health behavior results of single-factor analysis. No differences were observed in the variables of gargling after dinner, eating snacks, and eating sweet foods before sleeping. Crude OR's show that children who had visited a dentist in the preceding 12 months (OR = 0.4, *p* < 0.05, 95% CI = 0.3–0.6) and had a higher frequency of toothbrushing (OR = 0.5, *p* < 0.05, 95% CI = 0.4–0.8) had a decreased likelihood of caries.

**Table 2:** Single-factor analysis of the relationship between sociodemographic characteristics and caries experience in children with intellectual disabilities.

Characteristics	DMFT > 0 (*N* = 241)	DMFT = 0 (*N* = 209)	Unadjusted OR (95% CI) ^a^		*p* ^b^
	*N* (%)				
Gender					
Male	151 (62.7)	157 (75.1)	1.0		
Female	90 (37.3)	52 (24.9)	1.8	(1.2–2.7)	0.005
Father’s education level					
Low	50 (20.7)	42 (20.1)	1.0		
Medium	129 (53.5)	119 (56.9)	1.1	(0.7–1.8)	0.716
High	62 (25.7)	48 (23.0)	0.9	(0.5–1.6)	0.778
Mother’s education level					
Low	79 (32.8)	58 (27.8)	1.0		
Medium	130 (53.9)	125 (59.8)	1.3	(0.9–2.0)	0.243
High	32 (13.3)	26 (12.4)	1.1	(0.6–2.1)	0.754
Domestic economic status					
Low-income	15 (6.2)	10 (4.8)	1.0		
Middle-income	168 (69.7)	144 (68.9)	0.8	(0.3–1.8)	0.552
High-income	58 (24.1)	55 (26.3)	0.7	(0.3–1.7)	0.432
Registered residence					
Countryside	36 (14.9)	24 (11.5)	1.0		
City	205 (85.1)	185 (88.5)	0.7	(0.4–1.3)	0.282
School district **^c^**					
Liwan	39 (16.2)	41 (19.6)	1.0		
Hizhu	46 (19.1)	43 (20.6)	1.1	(0.6–2.1)	0.703
Yuexiu	47 (19.5)	46 (22.0)	1.1	(0.6–2.0)	0.815
Tianhe	55 (22.8)	35 (16.7)	1.7	(0.9–3.0)	0.106
Panyu	54 (22.4)	44 (21.1)	1.3	(0.7–2.3)	0.399
Grade of intellectual disability					
I	17 (7.1)	16 (7.7)	1.0		
II	40 (16.6)	42 (20.1)	0.9	(0.4–2.0)	0.791
III	105 (43.6)	77 (36.8)	1.3	(0.6–2.7)	0.510
IV	79 (32.8)	74 (35.4)	1.0	(0.5–2.1)	0.990
With cerebral palsy					
No	146 (60.6)	149 (71.3)	1.0		
Yes	95 (39.4)	60 (28.7)	1.6	(1.1–2.4)	0.017

Notes: **^a^** OR (95% CI) = odds ratio (95% confidence interval); **^b^** χ^2^-test; *p* < 0.05 is considered statistically significant; DMFT: Decayed, Missing due to caries, and Filled Teeth; **^c^** School district is defined as dummy viable, and Liwan is the reference cell, which is compared with all other districts.

**Table 3:** Single-factor analysis of the relationship between oral health behaviors and caries experience in children with intellectual disabilities.

Variables	DMFT > 0 (*N* = 241)	DMFT = 0 (*N* = 209)	Unadjusted OR (95% CI) ^a^		*p* ^b^
	*N* (%)				
Gargling after dinner					
No	189 (78.4)	152 (72.7)	1.0		
Yes	52 (21.6)	57 (27.3)	0.7	(0.5–1.1)	0.160
Dental visit in preceding 12 months					
No	202 (83.8)	142 (67.9)	1.0		
Yes	39 (16.2)	67 (32.1)	0.4	(0.3–0.6)	<0.001
Frequency of toothbrushing					
≤Once a day	179 (74.2)	127 (60.8)	1.0		
>Once a day	62 (25.7)	82 (39.2)	0.5	(0.4–0.8)	0.002
Eating snacks					
Never or seldom	45 (18.7)	42 (20.1)	1.0		
Occasionally or often	196 (81.3)	167 (80.0)	0.9	(0.6–1.5)	0.703
Eating sweet foods before sleeping					
Never or seldom	39 (16.2)	26 (12.4)	1.0		
Occasionally or often	202 (83.8)	183 (87.6)	0.7	(0.4–1.3)	0.260

Notes: **^a^** OR (95% CI) = odds ratio (95% confidence interval); **^b^** χ^2^-test; *p* < 0.05 is considered statistically significant; DMFT: Decayed, Missing due to caries, and Filled Teeth.

### 3.3. Results of the Multivariate Analysis

Results of the multivariate logistic regression analysis are shown in Table 4, which presented an adequate fit (goodness of fit, Hosmer and Lemeshow: χ^2^ (7) = 6.28, *p* = 0.508). The prevalence of caries was significantly related to some socio-demographic characteristics and oral health behaviors. The odds for girls having caries (DMFT > 0) were 1.9 times more than for boys. The odds for children with cerebral palsy having caries were 1.6 times more than for those without cerebral palsy. Having dental visits in the preceding 12 months (adjusted OR = 0.4, *p* < 0.05, 95% CI = 0.2–0.6) and toothbrushing at least twice a day (adjusted OR = 0.5, *p* < 0.05, 95% CI = 0.3–0.8) were caries-protective factors.

**Table 4:** Results of the multivariate logistic regression analysis for the relationship between risk/protective factors and caries experience in children with intellectual disabilities.

Variation	Adjusted OR (95% CI) ^a^		*p* ^b^
Gender			
Male	1.0		
Female	1.9	(1.2–2.8)	0.004
With cerebral palsy			
No	1.0		
Yes	1.6	(1.1–2.4)	0.030
Dental visit in the preceding 12 months			
No	1.0		
Yes	0.4	(0.2–0.6)	<0.001
Frequency of toothbrushing			
≤Once a day	1.0		
>Once a day	0.5	(0.3–0.8)	0.001

Notes: **^a^** OR (95% CI) = odds ratio (95% confidence interval); **^b^** Logistic regression; *p* < 0.05 is considered statistically significant; DMFT: Decayed, Missing due to caries, and Filled Teeth; Each variable was adjusted for all other variables in the final model.

## 4. Discussion

Nationwide oral epidemiology investigations were conducted in 1983, 1995, and 2005, but they included little, if any, information about the oral health status of IDC in China. The present study demonstrated that caries prevalence rates and DMFT in IDC are 53.5% and 1.5, respectively, in Guangzhou, which are well above the national index for 12-year-old children without disabilities (28.9% and 0.5) [22] and higher than those of teenagers with CP in Hong Kong (43% and 1.2) [23] . Our conclusion supported previous results [5,12,13,24] showing that the status of dental health in patients with disabilities was poorer compared with that of the general population, but is in contrast to the study done by Sagheri [25], which demonstrated that the prevalence of dental caries among preschool children with disabilities in Ireland is lower than that of the comparable general population. Well-established oral health preventive practices in Ireland may be related to the reduction in the prevalence of dental caries in children with disabilities. In addition, the prevalence of caries and DMFT varied among different types of disabilities. For example, Reddy and Sharma [26] reported the prevalence of caries and DMFT/dmft to be 40.0% and 1.1/0.2, respectively, in visually impaired children, and 11.5% and 0.9/0.5 in children without visual impairment. Jaber [8] reported that children with autism exhibited a higher caries prevalence (77.0%) and DMFT (1.6) than did children in a non-autistic healthy control group (caries prevalence = 46%, DMFT = 0.6).

This study also demonstrated that the odds ratios for IDC with CP were significantly higher than for those without CP, supporting the results of previous studies [12]. The increased risk of caries experience in IDC with CP might be related to complications (visual impairments, hearing impairment, seizures, and motor impairment, *etc.*), that could lower their physical abilities and be barriers to adequate oral care [5]. Researchers also reported that CP would lead to abnormal movements of the tongue and facial muscles [27] as well as low salivary flow, pH, and buffering capacity [28], which might increase the risk for dental caries. In addition, medication may contribute to complications. For example, there can be various neurologic and metabolic syndromes in children with CP, some involving seizures [29] and the medication administered to control seizures in children is frequently laced with sugars to make it more palatable. The medication then reduces salivary flow, making the child more vulnerable to dental decay [30]. Moreover, children with CP may have reduced control of gastrointestinal mobility, which reduces food intake and may result in malnutrition [31]. These children often require high calorie supplementary feeding to maintain body weight, and these supplements are high in extrinsic sugars which have a deleterious effect on oral health [32]. It is not surprising therefore that children with CP may have reduced levels of oral health.

Routinely visiting the dentist and toothbrushing are considered to be caries-preventive measures [33,34,35]. In our study, only 23.6% of IDC had visited a dentist in the preceding 12 months and 32% of IDC brush their teeth more than once a day; their prevalence rates for caries and DMFT scores were significantly lower in comparison with those of other children (*p* < 0.01). In China, because of a lack of dentists [36] and insufficient programs for public information and education about oral health, many parents have little access to information on dental health [37], and parental lack of scientific understanding can result in low rates of dental consultation for IDC faced with caries lesions [9,38]. Further, IDC always have difficulties in self-cleaning and communicating oral health needs [39]. They also cannot understand the significance of oral cleanliness, and tend not to cooperate and even resist toothbrushing, which is another barrier to parental success in helping their children with oral hygiene [5]. Toothbrushing as an effective method for the mechanical removal of bacterial biofilm [40] is even more important for these children [41]. The results of our study concluded that brushing twice a day helps to lower the prevalence of dental caries. The odds ratios for children who brushed their teeth twice a day were significantly lower than for those who brushed their teeth daily or less often, with an odds ratio of 0.5 (95% CI = 0.3–0.8). We recommend that the likelihood of dental caries prevention be strengthened by improving the frequency of toothbrushing to at least twice a day for IDC. Therefore, there is an urgent need for the government to take actively oral health promotion measures, intervening in the oral health of IDC. According to the WHO [42], building healthy public policy is important for policy-makers. In the early 21st century, the American Academy of Pediatrics developed the concept of a “dental home”, which means “an ongoing relationship between the dentist and the patient, inclusive of all aspects of oral health care delivered in a comprehensive, continuously accessible, coordinated, and family-centered way” [25]. Chinese policy makers may learn from this concept to establish a “Chinese dental home”. Due to the lack of dentists, the cultivation of a community-based dentist is now a top priority for the government, which can improve the children’s access to dental care and address the above barriers to dental services for IDC. In addition, a supportive environment and comprehensive oral health educational programs for parents are also imperative, to improve parental skills in maintaining the oral health of their children and provide an important basis for enhancing the role of parents in preventing dental caries among IDC.

Fewer DMFT scores were found for males than for females in this study, which was similar to the study done by Yee and Altun [5,43], while in contrast to findings of Sachin [44]. Chinese patriarchal traditions might be a reason that parents with male children were likely to pay more attention to their health compared with that of their female children. This requires further study.

## 5. Conclusions

The results of this study show poorer dental health status in IDC, who have higher caries prevalence rates and DMFT scores, and enhance our understanding of the role that oral health behaviors play in the dental health status of IDC in Guangzhou, China. IDC with CP have a higher risk of caries experience than IDC without CP, while toothbrushing more than twice a day and routine dental visits are caries-protective factors. These results indicate that improving the IDC’s access to dental care and comprehensive oral health educational programs for parents are urgently needed, which may lead to a reduction in dental caries levels in IDC.
